# Liquids as Reinforcements for Anisotropic and Tough Soft Matter Composites

**DOI:** 10.1002/adma.72447

**Published:** 2026-02-07

**Authors:** Gwyneth M. Schloer, Ohnyoung Hur, Ravi Tutika, Aaron Haake, Eric J. Markvicka, Michael D. Bartlett

**Affiliations:** ^1^ Mechanical Engineering Soft Materials and Structures Lab, Virginia Tech Blacksburg Virginia USA; ^2^ Macromolecules Innovation Institute Virginia Tech Blacksburg Virginia USA; ^3^ Mechanical & Materials Engineering Smart Materials & Robotics Lab University of Nebraska–Lincoln Lincoln Nebraska USA; ^4^ Electrical & Computer Engineering University of Nebraska–Lincoln Lincoln Nebraska USA; ^5^ School of Computing University of Nebraska–Lincoln Lincoln Nebraska USA

**Keywords:** all‐soft composite, crack steering, liquid metal, mechanical anisotropy

## Abstract

Biological tissues and engineered composites achieve exceptional mechanical properties through microstructures that create stiffness and toughness in preferred directions. While composites traditionally leverage solid reinforcements to drive this anisotropy, directional mechanics in all‐soft matter composites remain a longstanding challenge, despite their importance for soft devices that stretch and adapt under load. Here, we create all‐soft matter composites where liquid inclusions direct and enable anisotropic and heterogeneous mechanical properties. By shaping and orienting liquid metal droplets within elastomers, we program directional stiffness, enhance toughness beyond 36,000 J m^−2^, and guide cracks along non‐linear paths with deflections up to 150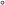 during extreme deformations. This allows liquids, which are up to a million times softer than traditional rigid inclusions, to act as mechanical reinforcements. These liquid inclusions enhance directional stiffness or softness relative to unfilled elastomers and enable programmable crack‐path engineering that surpasses simple blunting or trapping, with anisotropy tuned on demand during processing. We leverage this to protect soft circuits even under catastrophic damage, offering new possibilities to direct mechanical forces in compliant materials for resilient soft electronics and robots, wearables, and morphing matter.

## Graphical abstract

Liquid metal droplets are shaped and oriented within soft elastomers to create all‐soft matter composites with programmable mechanical anisotropy. These liquid inclusions act as fiber‐like reinforcements, enabling directional stiffness, enhanced toughness, and controlled crack steering under extreme deformation, offering new routes to resilient soft electronics, robotics, and morphing materials.

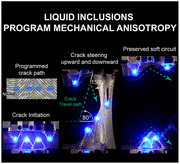

## 1 Introduction

Anisotropic composites with directionally dependent mechanical properties are leveraged by nature and engineered systems to achieve exceptional properties and performance [1, 2, 3, 4]. In these diverse systems, anisotropy arises due to the controlled distribution and orientation of solid, rigid inclusions or molecules. This imparts tailored properties such as stiffness, strength, or toughness in specific orientations and in desired locations, greatly increasing the design space and utilization of materials [5, 6, 7, 8, 9]. This directionality enables the efficient use of inclusions in natural systems like wood [10], bones [11], and corals [12], and has been critical for diverse applications in synthetic materials like carbon‐fiber reinforced plastics [13], and reinforced concrete [14]. In these anisotropic composites, the inclusion is typically stiffer than the matrix material, both phases are solids, and the composite experiences limited deformation during use (i.e., load bearing applications).

Recently, all‐soft matter systems composed of highly deformable materials such as rubbers, gels, and fluids have emerged [15, 16, 17, 18]. These materials offer unique capabilities beyond those of rigid‐inclusion composites [19], and are essential for applications in soft electronics and robotics, wearables, and morphing matter [20, 21]. Biological tissues like muscle and tendon exemplify this concept, achieving directional stiffness and toughness through structures that adapt to mechanical loads, enabling directional force transmission and promoting crack deflection [22, 23, 24]. Inspired by these natural systems, synthetic soft materials have been developed to exploit liquid and polymer networks for enhanced mechanical and functional properties. For example, polymer gels with cross‐linked polymer molecules swollen in liquids exhibit remarkable toughness and stretchability [25, 26, 27], while liquid droplets embedded in soft solids enable functionalities such as optical tuning, electrical conductivity, and actuation [28, 29]. However, achieving mechanical anisotropy in soft materials using liquid inclusions presents two challenges. First, liquid droplets naturally form spheres to minimize surface energy, making them difficult to shape and elongate, which results in isotropic properties [30, 31]. Second, liquid droplets are typically distributed homogeneously, limiting spatial control over mechanical properties [32]. As a result, most liquid droplet‐solid composites lack directional mechanical properties seen in biological tissues and anisotropic engineered composites.

**FIGURE 1:**
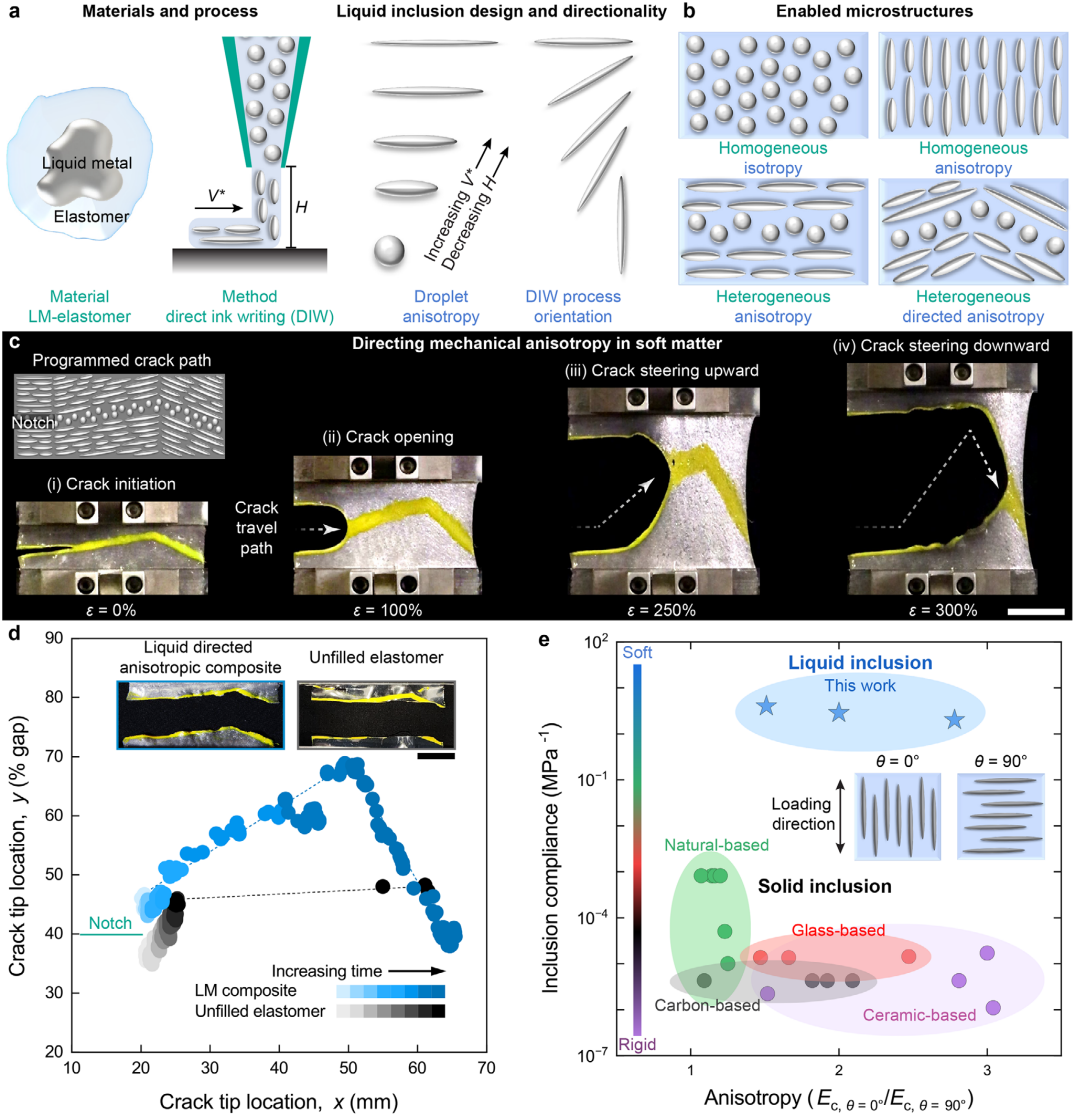
Directing mechanical anisotropy in soft matter with liquid inclusions. (a) Schematics of material and process strategies to tune liquid metal composite droplet morphology. (b) Distinct liquid inclusion microstructures created in soft matter composites, from homogeneous and isotropic to heterogeneous with directed anisotropy. (c) Programmed crack path in soft matter guided by tailored liquid inclusions; the crack follows a precise, inclusion‐directed non‐linear trajectory (yellow trace). Scale bar is 20 mm. (d) Crack tip location over time with lateral (x‐axis) and longitudinal (y‐axis) progression (y‐axis represented as the % gap between grips). Inset shows LM composite and unfilled elastomer samples after crack propagation. Scale bar for images is 20 mm. (e) These liquid inclusion composites define a new region in an Ashby‐style map of mechanical anisotropy in soft, short‐fiber systems.

Here, we introduce all‐soft matter composites where liquid inclusions direct and enable tunable anisotropic and heterogeneous mechanical properties. Using direct ink writing (DIW), we shape, elongate, and orient liquid metal (LM) droplets dispersed in soft elastomers, creating morphologies that range from homogeneous to heterogeneous and isotropic to anisotropic, enabling designer mechanical properties (Figure 1a,b). We find that these liquid droplets direct the mechanical response in soft matter, enabling programmable directional stiffness, resisting deformation in specific orientations, and guiding cracks along defined, non‐linear paths. Counter intuitively, the LM droplets behave like solid‐inclusions in short fiber composites (i.e., carbon fiber or glass fiber), enabling these orientation specific mechanical behaviors, unlike isotropic liquid droplet composites [32]. We then leverage directed anisotropy, defined as heterogeneously orientated anisotropic structures, to fabricate LM composites with both spherical and elongated droplet microstructures to systematically control fracture properties (Figure 1c; Movie S1). This enables dramatic crack path steering in highly extensible systems, where cracks follow a prescribed non‐linear trajectory dictated by LM droplet morphology (indicated by yellow dye), in contrast to a conventional linear crack path in pristine elastomers (Figure 1d; Movie S2). As shown in Figure 1e, these soft LM composites achieve mechanical anisotropy ratios ($E_{c,\;\theta \;=\;0^{\circ }}$/$E_{c,\;\theta \;=\;90^{\circ }}$) comparable to soft composites with solid short‐fibers (ceramic [33, 34], carbon [35, 36, 37], glass [13, 38], and natural [39, 40, 41, 42]), yet the LM inclusions remain up to six orders of magnitude more compliant than the solid inclusions, opening an entirely new design space. Directed anisotropy and heterogeneity, achieved through programmed liquid inclusions in solids, unlocks emergent anisotropic mechanical responses for soft matter, advancing resilient, multifunctional systems for applications in electronics, robotics, and morphing devices.

## 2 Results and Discussion

### 2.1 Anisotropic Mechanical Properties in LM Composites

The composites consist of LM at 50% by volume in a silicone matrix. By using different polymers to vary the modulus of the composite systems, all inks exhibited shear‐thinning behavior, which enabled the use of the DIW process (Figure S1). The LM droplet microstructures are fabricated through a DIW process where print parameters are tuned to locally control the LM microstructure [43, 44]. To control the LM droplet shape, the process parameters of interest are the non‐dimensionalized nozzle velocity ($V^{*}$ = V/C), which represents the ratio of the nozzle velocity (V) to extrusion velocity (C), and the nozzle height (H) above the print surface. By increasing $V^{*}$ and decreasing H during printing, the spherical LM droplets in the initial ink are elongated to ellipsoidal shapes in the printing direction. Unlike conventional fluid droplets, LM droplets elongate into stable ellipsoids due to a surface oxide. Here, $V^{*}$ = 2 and $V^{*}$ = 12 are used to create generally spherical drops and elongated LM droplets, respectively. Unfilled elastomer, without LM inclusions, is also printed under the same conditions.

**FIGURE 2:**
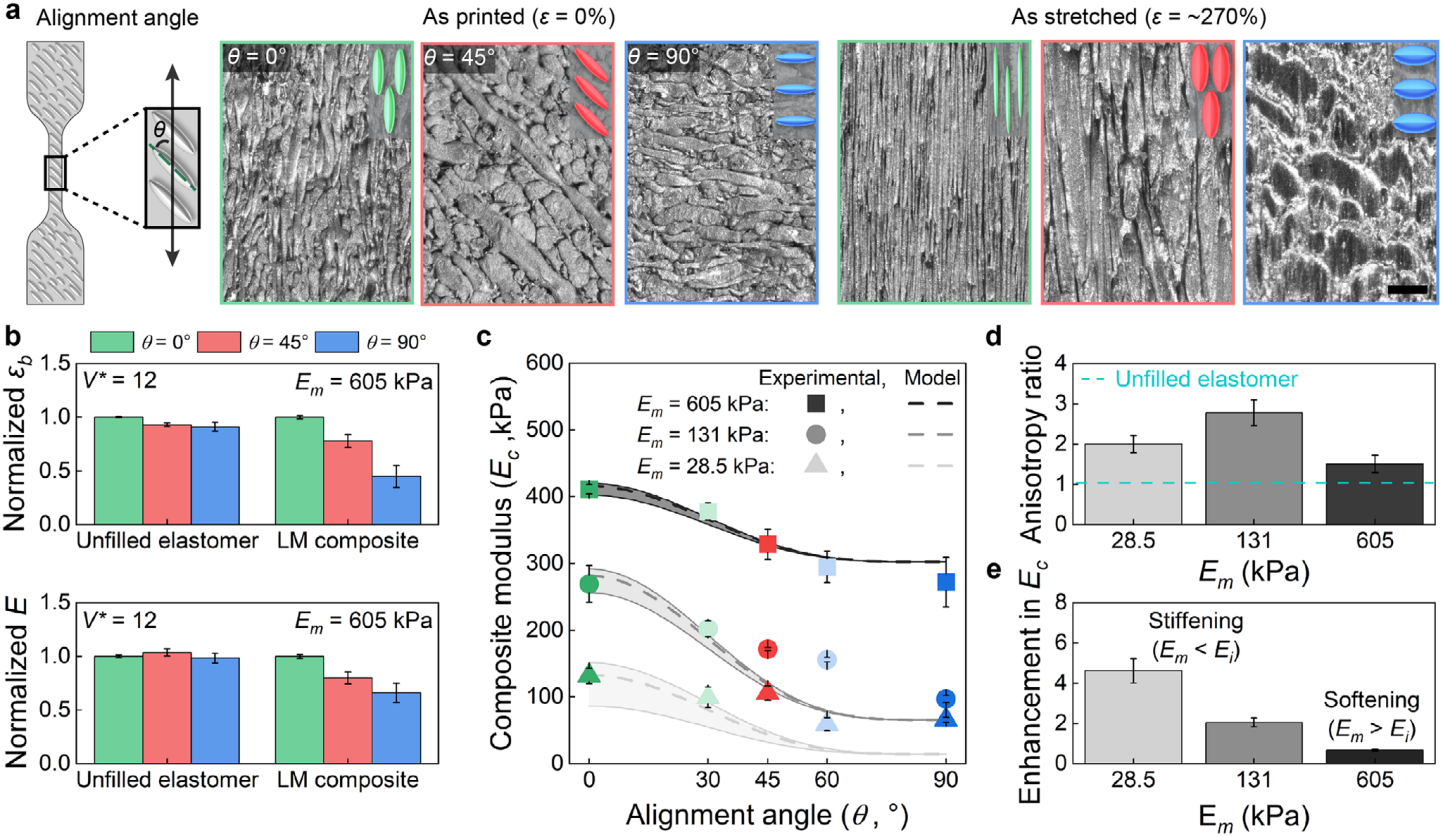
Mechanical anisotropy in liquid inclusion composites. (a) Tensile sample schematic showing the loading axis (black arrow) and droplet major axis (green dashed line); θ indicates the angle between them. Optical micrographs of LM composite ($V^{*}$ = 12) samples with elongated droplets oriented at θ = 0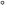, 45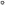, 90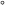 at ε = 0% (left), ∼ 270% (right). Scale bar is 200 μm. (b) Normalized strain at break ($\varepsilon_{b}$) and tensile modulus (E) calculated from stress–strain curves. The values are normalized with respect to the values at θ = 0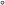. (c) Cox‐Krenchel model fits on the experimental data to model the tensile modulus as a function of the LM droplet orientation (θ). The shaded area represents the standard deviation (SD) of LM droplet aspect ratio. (d) Mechanical anisotropy ratio ($E_{c,\;\theta \;=\;0^{\circ }}$/$E_{c,\;\theta \;=\;90^{\circ }}$) for different $E_{m}$, where the dash line represents unfilled elastomer. (e) Enhancement in composite modulus ($E_{c}$) presented as a ratio of LM composite modulus at 0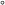 to unfilled elastomer at 0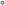. Data are presented as mean ± 1 s.d. (n = 3).

To determine the effect of the liquid droplet alignment direction on mechanical properties, samples with different droplet orientations are tested under uniaxial tension. Orientation is controlled by the DIW printing direction and characterized by θ, which is the angle between the elongated droplet's major axis and the loading axis (Figure 2a schematic). For unfilled elastomer samples, θ, denotes the angle between the printing direction and the loading axis. Microscopy images of as‐fabricated samples (ε = 0%) with three different orientations of θ = 0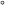, 45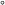, and 90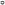 are presented in Figure 2a. These samples are then subjected to a uniaxial tensile strain (ε), which resulted in deformation of the droplets as seen in the in situ microscopy images in Figure 2a. For θ = 0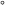 droplets, strain further elongates the LM droplets into fiber‐like shapes. At θ = 45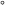, droplets reorient toward the loading axis while increasing in aspect ratio. In θ = 90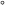 samples, droplets remain perpendicular to the load, stretching along their minor axis and reducing their aspect ratio.

The mechanical durability of the LM soft composites was evaluated via cyclic uniaxial tensile tests for 100 cycles at strain amplitudes up to 300% (Figure S2a,b). Both $\theta =0^{\circ }$ and $\theta =90^{\circ }$ orientations exhibit characteristic hysteresis in the initial loading cycle followed by a repeatable, steady‐state mechanical response with little hysteresis, representative of the Mullins effect in filled elastomer systems. This macroscopic stability is corroborated by the microstructural integrity shown in Figure S2c,d. After 100 cycles, the $\theta =0^{\circ }$ samples show negligible changes, retaining their fiber‐like morphology. Similarly, the $\theta =90^{\circ }$ samples maintain their transverse orientation without notable irreversible distortion. Creep and stress relaxation experiments were also performed in the $\theta =0^{\circ }$ and $\theta =90^{\circ }$ orientations and in the unfilled system, showing viscoelastic behavior typical of polymers (Figure S3). These results confirm that the programmed LM architectures are mechanically robust and maintain their structural integrity throughout repetitive loading‐unloading cycles.

Mechanical properties including strain at break ($\varepsilon_{b}$) and the tensile modulus (E) are quantified from the uniaxial stress–strain curves (Figure S4). The data is presented in Figure 2b, which is normalized for each sample set relative to the θ = 0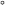 data. The unfilled elastomer samples without LM droplets do not show a dependence of strain at break or modulus relative to the print direction (θ), showing isotropic properties (Figure S5). Meanwhile, the LM composites with oriented LM droplets printed at $V^{*}$ = 12 show a strong dependence on θ, where $\varepsilon_{b}$ and E both decrease as θ is increased (Figure 2b). For more spherical LM droplets ($V^{*}$ = 2), minimal property changes are measured, indicating generally isotropic properties, with a small degree of anisotropy due to some LM droplet orientation (Figure S6). Taken together, these results show minimal influence of elastomer orientation on mechanical properties, as the unfilled elastomer is isotropic, whereas the elongated LM droplets show pronounced anisotropic mechanical properties due to the droplet shape and orientation.

To model the anisotropic mechanical properties of the elongated LM droplets, we leverage theories originally developed for rigid, short fiber composites. The elongated LM droplets have similar geometric features to short fiber composites, including high aspect ratios and orientation of the inclusions. Following the Cox‐Krenchel model modified by Carman‐Reifsnider [45], the elastic modulus of a short fiber composite, $E_{c}$, can be described as:

$$E_{c}=E_{i}\;\eta_{0}\;\eta_{l}\;\phi_{i}+E_{m}\left(1-\phi_{i}\right)$$

where $E_{i}$ and $E_{m}$ are the elastic moduli of the inclusion and the matrix, and $\phi_{i}$ is the volume loading of fiber inclusions. $\eta_{l}$ is the Cox fiber‐length efficiency factor which takes into account the aspect ratio (AR) and relative material properties ($E_{i},E_{m}$), and $\eta_{0}$ is the Krenchel orientation factor which considers inclusion orientation (see Supporting Information for more details).

Figure 2c shows model fits to experimental composite modulus data for three different matrix materials distinguished by their matrix modulus $E_{m}$. In each case, $E_{m}$ and AR are experimentally measured, while the $E_{i}$ is used as a fitting parameter. For the stiffest matrix material (square symbol), $E_{m}$ = 605 kPa and AR = 4.90 (Figures S5 and S7) while $E_{i}$ = 250 kPa is set as a fitting parameter. The model agrees well with data for elongated LM droplets printed at $V^{*}$ = 12 for five orientations (θ = 0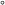, 30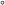, 45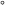, 60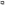, and 90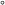), where composite modulus is highest at low θ and decreases as θ increases (see Figure S8 for statistical analysis of orientation angle). For an intermediate modulus matrix (circle symbol), $E_{m}$ = 131 kPa, AR = 9.8, and $E_{i}$ = 500 kPa; for the softest matrix material (triangle symbol), $E_{m}$ = 28.5 kPa, AR = 7.4, and $E_{i}$ = 350 kPa. The model matches both datasets for θ = 0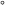 and 30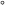, but diverges for θ > 30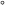. This deviation is attributed to limitations in the model, which predicts the modulus at θ = 90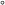 to be half the matrix modulus, neglecting the inclusion contribution, which may be reasonable for inclusions, which are orders of magnitude more rigid than the matrix, but for our system, the inclusion is on the order of the matrix modulus. Overall, the trend is captured well and shows that the modulus of the oriented composite decreases as the orientation angle increases. In contrast, samples with spherical droplets (AR≈1.7, Figure S9) printed at $V^{*}$ = 2 show minimal dependence of elastic modulus on orientation angle (Figure S10), which is consistent with predictions for randomly distributed spherical inclusions.

Although fluid droplets are typically thought to soften composites [32], the elongated and oriented LM droplets exhibit unusual behavior by directing mechanical anisotropy and stiffening the composite when aligned with the loading direction (low θ). This behavior is notable, whereas rigid‐fiber systems typically rely on inclusions orders of magnitude stiffer than the matrix [46] (Figure 1e), LM droplets can be comparable in stiffness, or even softer, yet still direct strong anisotropic reinforcement (Figure S11). Their ability to do so arises from a combination of the surface oxide shell and surface tension [47, 48], which together impart an effective solid‐like modulus. Critically, elongation of the droplets amplifies these effects by increasing the surface‐to‐volume ratio, enhancing resistance to deformation in the loading direction. This unique coupling of fluid inclusions, interfacial mechanics, and droplet geometry distinguishes LM composites from both conventional liquid‐filled systems and rigid‐fiber composites.

This anisotropic response mimics short‐fiber composites, where alignment of stiff fibers such as cellulose in hydrogels or glass fibers in elastomers increases modulus at θ = 0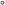 relative to θ = 90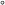 [40, 49, 50]. Similarly, LM composites exhibit notable anisotropy, with modulus at θ = 0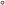 exceeding that at θ = 90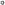, demonstrating that LM droplets can display a fiber‐like mechanical response (Figure 2d). Furthermore, as ϕ was varied, the mechanical anisotropy exhibited a corresponding shift; it increased steadily up to ϕ=50% and subsequently underwent a slight decrease beyond ϕ=50% (Figure S12). The anisotropy ratios achieved in our soft LM composites are comparable to those of rigid short‐fiber systems (ceramic, carbon, glass, and natural fibers), yet the LM inclusions remain up to six orders of magnitude more compliant than their rigid‐inclusion counterparts. This expands the design space for soft matter systems, enabling anisotropic reinforcement with highly compliant inclusions.

Finally, the overall composite stiffening ($E_{c,\;\theta \;=\;0^{\circ }}$/$E_{m}$) exceeds other examples with liquid inclusions. While glycerol droplets in elastomers stiffen the composite by 30% [32], elongated LM droplets can increase modulus by up to ∼460% (Figure 2e). This is also tunable depending on the relative stiffness of the matrix ($E_{m}$) and inclusion ($E_{i}$), LM droplets either stiffen ($E_{m}<E_{i}$) or soften ($E_{m}>E_{i}$) the composite. Thus, orientated and aligned LM droplets provide a unique approach to tune stiffness and anisotropy in soft composites, unlocking new opportunities for programming mechanical response in soft matter systems.

### 2.2 Crack Propagation Behavior of Anisotropic Liquid Metal Composites

Programmed mechanical response in materials has been leveraged to control crack propagation in metals, alloys, and anisotropic solids [51, 52, 53, 54]. In rigid systems like metals, dissimilarities such as defects and grain boundaries can be created or employed to control a crack path [55, 56]. However, with the emergence of devices and applications that employ soft polymeric materials or hybrids of rigid and soft materials, the control of failure and specifically tuning crack behavior has become increasingly important [57].

To investigate how anisotropic LM droplets influence fracture behavior, we used LM composites based on an elastomer with a $E_{m}$ of 605 kPa and conducted pure shear fracture tests on samples with three LM droplet configurations: (1) isotropic spherical LM droplets ($V^{*}$ = 2), (2) anisotropic elongated LM droplets oriented perpendicular to stretch ($V^{*}$ = 12, θ = 90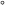), and (3) anisotropic elongated LM droplets aligned with the stretching direction ($V^{*}$ = 12, θ = 0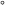). Each sample was notched and then stretched to drive crack propagation. Throughout the experiment, crack progression was tracked by monitoring the crack tip position. This allows us to quantify the effects of droplet shape and orientation on crack propagation (cracks traveling horizontally across the sample) and crack deflection (cracks traveling vertically in the loading direction).

**FIGURE 3:**
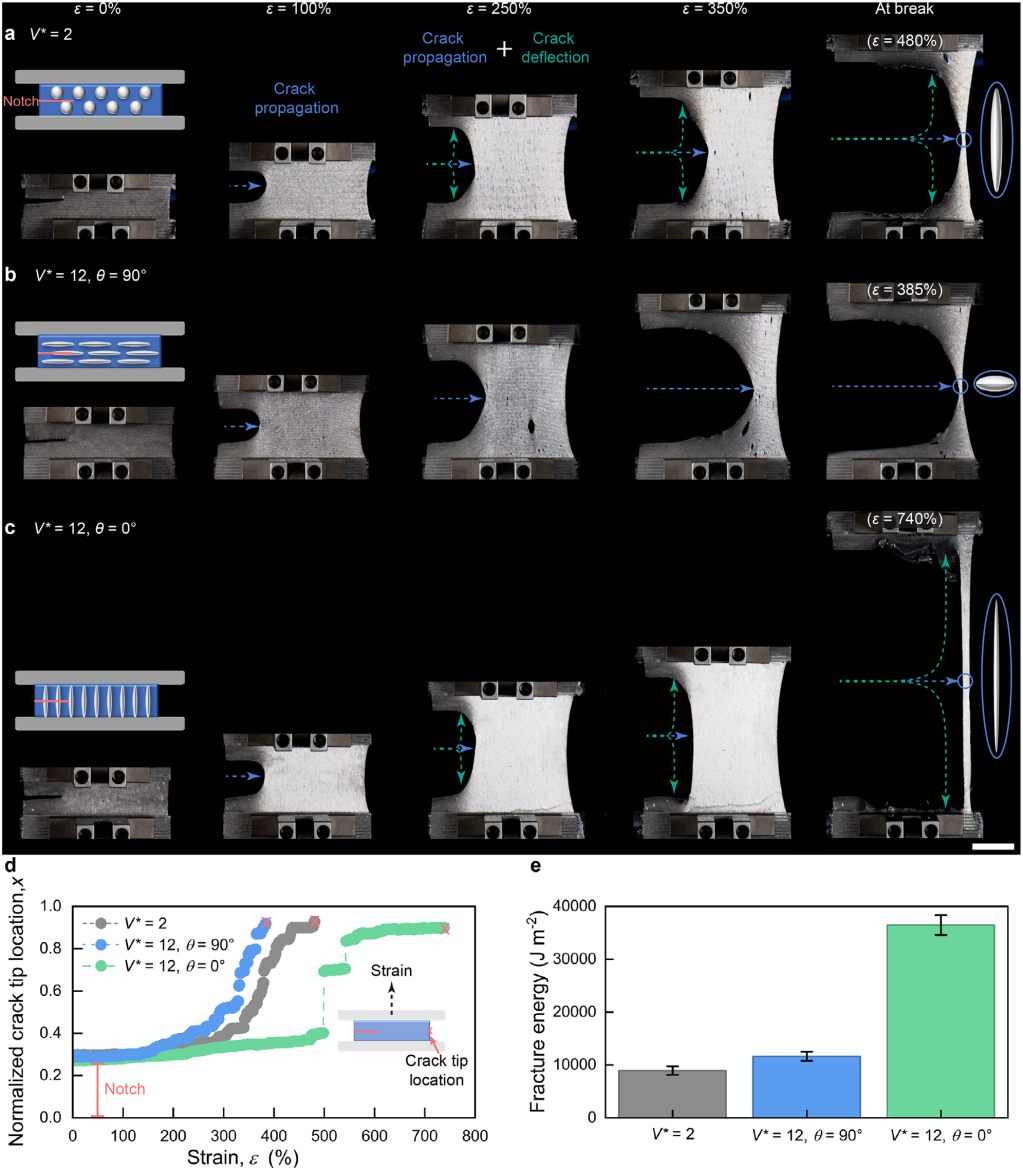
Crack propagation behavior in liquid inclusion composites. Pure shear test images: (a) Isotropic sample with spherical LM droplets showing crack propagation followed by deflection. (b) Anisotropic sample with θ = 90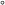 elongated LM droplets showing crack propagation through the material without any deflection. (c) Anisotropic sample with θ = 0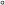 elongated LM droplets showing almost immediate crack deflection. Scale bar is 20 mm. (d) Normalized crack tip lateral progression (normalized by initial sample width) as a function of strain. (e) Fracture energy of LM composites with different microstructures. Data are presented as mean ± 1 s.d. (n = 3).

Figure 3a–c and Movie S3 show the samples during several phases of loading, from undeformed until immediately before the failure strain. In addition to the as‐fabricated programmed droplet shape, stretching causes further droplet deformation, as observed in Figure 2a. For example, isotropic LM droplets from cast samples have been shown to blunt and deflect cracks, which causes propagation in the direction of stretch, instead of propagating across the sample as expected [58]. In the printed isotropic sample, LM droplets also elongated with strain, causing initial crack deflection but ultimately allowing the crack to propagate across the sample and fail at a strain of 480% (Figure 3a). In samples with θ = 90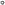 droplets, LM inclusions guided the crack horizontally and accelerated crack propagation during strain (Figure 3b). This resulted in failure at a comparatively lower strain of 385%. Conversely, for samples with θ = 0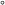 droplets, pre‐elongated LM droplets further stretched under load, leading to notable crack blunting (Figure 3c). The crack tip was rapidly eliminated compared to the other samples, increasing resistance to propagation, and enabling the highest strain of 740% before failure.

Tracking crack progression revealed that θ = 90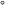 droplets promoted the fastest failure, while θ = 0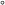 droplets showed the greatest crack deflection and blunting, offering the highest resistance to propagation. The isotropic sample displayed intermediate behavior (Figure 3d). Notably, once the crack begins to propagate in the θ = 90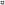 and isotropic samples, cracks progressed continuously, with slight arrests periodically. However, for the θ = 0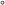 sample, cracks were arrested for large extents of deformation and then moved in steps, showing how the anisotropic droplets oriented perpendicular to the crack provided a large barrier for crack propagation. This increase in crack resistance can be measured as an increase in fracture toughness. The $\theta =0^{\circ }$ samples more effectively arrest crack tips and redirect mechanical energy, resulting in nearly a four‐fold increase in fracture energy, up to 36,460 ± 1,870 J m^−2^, compared to isotropic counterparts with the same LM loading created with $V^{*}$ = 2 (Figure 3e). The $\theta =90^{\circ }$ samples showed a toughness similar to the isotropic samples and less than $\theta =0^{\circ }$ samples, showing an orientation dependent toughness. In all three examples, the LM droplets are effectively able to tune the fracture process. Importantly, the liquid droplets in the anisotropic samples are able to notably impact the crack dynamics and toughness in highly extensible all‐soft matter composites beyond isotropic samples. In contrast to previous crack deflection work in rigid and solid composites, these results highlight how droplet anisotropy, both in shape and orientation, provides a means to tailor crack behavior in soft solids.

### 2.3 Designing Advantageous Failure in Soft Electronics

We find that the droplet orientation controls the morphology of the fracture surface when the samples fail under tension. As seen in Figure S13a, as θ increases from 0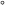 to 90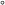, the angle of the fracture surface increases to follow the droplet orientation. For 0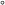, the fracture surface is perpendicular to the loading direction and shows little orientation as the droplets do not have an angular component. In Figure S13b, the droplet alignment and fracture surface angle are well correlated to each other and to the designed droplet orientation θ. Meanwhile, the fracture surface is consistently perpendicular to the loading direction for the unfilled elastomer without LM inclusions, despite different printing orientations relative to the loading direction (Figure S14). This shows that the LM inclusions are controlling the deformation and fracture behavior, while the printed elastomer has minimal impact on the mechanical response.

**FIGURE 4:**
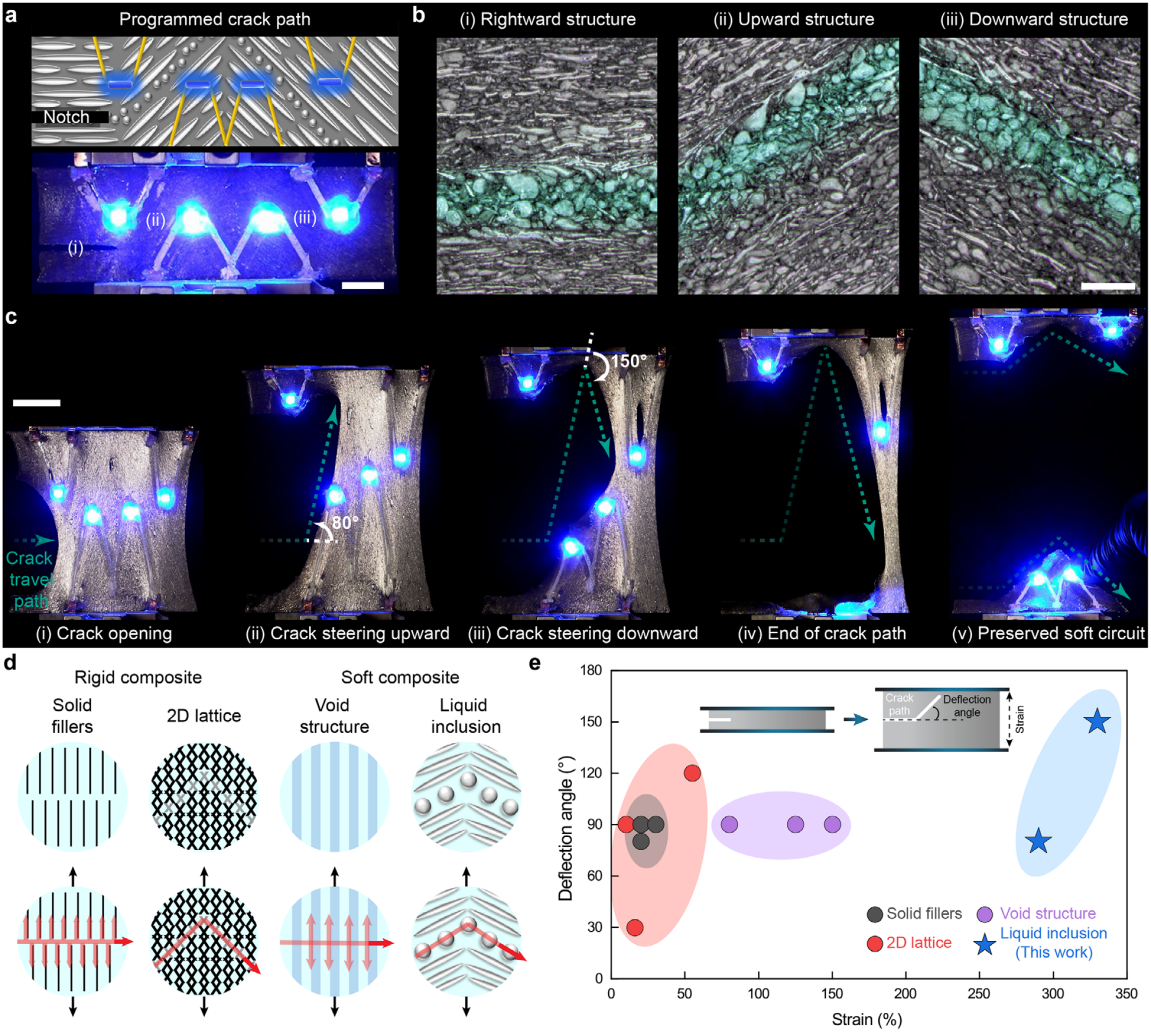
Directing fracture in soft electronics. (a) A schematic and image at 0% strain of an LM composite soft device programmed microstructures for a prescribed crack path. Scale bar is 10 mm. (b) Microscopy images of the programmed crack path in regions (i)‐(iii) from (a). The false color shows the spherical droplets in the designated crack path. Scale bar is 1 mm. (c) Image sequence (i–iv) showing crack propagation under uniaxial tension as the crack follows the programmed non‐linear path with extreme angular deflections that avoid the LEDs; (v) shows the intact circuit after full crack progression. Scale bar is 20 mm. (d) Schematics illustrate approaches to deflect/steer cracks in different composites systems. Shaded red lines show the crack trajectory; solid red arrows indicate end points. (e) Property map plotting deflection angle as a function of strain, with the schematic defining the angle. The liquid‐inclusion composites achieve both higher strains and larger crack‐deflection angles, highlighting their enhanced crack steering capability.

Leveraging this knowledge of LM droplet orientation for mechanical anisotropy and DIW for fabrication of parts with heterogeneous properties, we demonstrated programmable fracture paths to maintain continuous function of a highly deformable, soft circuit undergoing catastrophic failure. The soft circuit consisted of 4 LEDs in a horizontal line on a heterogeneous LM composite substrate, where DIW parameters ($V^{*}$ and H) control the placement of spherical and elongated LM droplets (Figure 4a). Uniaxial tension experiments show that cracks propagate through spherical droplets but are blunted by the major axis of elongated droplets (Figure 3). Using this principle, we design a crack path with spherical droplets (shaded with blue), flanked by elongated droplets to guide crack propagation around critical circuit components (Figure 4b). As the circuit is stretched, the crack follows the programmed path. First, progressing horizontally before steering upward at an $∼80^{\circ }$ angle and then steering again with a dramatic change in angle of $∼150^{\circ }$ in the downward direction while fully under load (Figure 4c i–iv; Movie S4). This ultimately splits the circuit into two sub‐circuits without any electrical failure, as demonstrated by the continuous illumination of the LEDs (Figure 4 cv). Notably, the crack remains on its designed path even when unintended material failure occurs (Figure 4 ciii, iv), demonstrating robustness of the approach. This experiment demonstrates the ability to precisely steer a crack undergoing extreme deformations through a designed path. In this example, the circuit remains functional despite catastrophic material failure, demonstrating a broader strategy for protecting critical components by programming fracture paths within highly deformable and soft matter systems.

This approach enables a soft composite system capable of dramatically steering a crack through a complex, pre‐designed path under large strains. Previous research has explored multiple strategies to control crack propagation in both rigid and soft composite systems (Figure 4d). In rigid systems, composites reinforced with solid fillers (e.g., fibers),[49, 59, 60] or architected 2D lattices,[51, 61] often fabricated through additive manufacturing, can deflect cracks but show minimal strain capacity due to their rigid nature. In soft composites, most approaches focus on crack blunting, including fabricating void structures or embedding modulus‐mismatched strips to arrest or divert a propagating crack [62, 63, 64]. However, these methods typically allow only modest crack deflection and lack the ability to precisely steer a crack along a prescribed, multi‐angle trajectory.

Our liquid inclusion‐based approach uniquely combines large deformation, enabled by the inherent softness of the matrix and deformability of the liquid inclusions, with programmed fracture paths achieved through processing to achieve microstructural anisotropy and heterogeneity. As a result, our fully soft composite system exhibits exceptional combinations of high crack‐deflection angles under large strains, surpassing both prior soft and rigid systems (Figure 4e). These capabilities transform fracture from an unavoidable failure mode into a programmable design feature, enabling enhanced robustness in soft materials and devices.

## 3 Conclusion

This work introduces an all‐soft composite with tunable anisotropic mechanical properties. By shaping, elongating, and orienting LM droplets, we control microstructures and mechanical behavior, leading to anisotropic mechanical properties that depend on droplet orientation. These liquid composites achieve anisotropy ratios similar to solid filler soft composites with liquid inclusions that are orders of magnitude more compliant and enhance the reinforcing effect (i.e., overall composite stiffening ($E_{c,\;\theta \;=\;0^{\circ }}$/$E_{m}$)) relative to other examples with liquid inclusions. We show that elongated droplets enable programmable stiffness, resist deformation in specific orientations, and act as mechanical guides that steer cracks along non‐linear, predefined paths. In effect, we utilize liquid droplets to direct mechanical properties in soft matter. We utilize this capability to demonstrate directed anisotropy by spatially patterning the orientation and shape of LM droplets to tailor local mechanical response, which gives rise to the ability to guide crack trajectories along engineered paths, enabling protection of critical components as a material undergoes catastrophic failure. This demonstrates a new capability for engineering resilient soft matter, offering versatile crack path control at strains far beyond the operational limits of conventional approaches. This approach unlocks a class of architected solid–liquid composites where microstructural anisotropy dictates macroscopic mechanical response. The ability to engineer anisotropic mechanical properties with different types of soft polymers and controlled fracture behavior mirrors key characteristics of soft biological tissues, such as muscle and tendon, which exhibit directional stiffness and toughness. Inspired by these natural systems, future work could explore bioinspired designs for adaptive soft materials in wearable electronics, robotics, and morphing structures that integrate mechanical resilience with multifunctionality.

## 4 Experimental Section

### 4.1 Fabrication and Printing of LM Ink

We used three different types of matrices: polydimethylsiloxane (PDMS, Sylgard 184 from Dow Inc.) with weight ratios of part A to part B of 40:1 and 60:1 and a two‐component silicone elastomer (ExSil‐100 from Gelest Inc.). Fumed silica (lateral size: 16 nm) was incorporated into only PDMS (10 wt.% to polymer) to modify its rheological properties and facilitate LM elongation. When using PDMS (60:1), silicone oil (AR 20, Sigma–Aldrich) was added to further soften the matrix. To fabricate the ExSil‐100 elastomer, parts A and B were combined in a 100:1 mass ratio. All polymers and additives were blended and degassed using a Flacktek Speedmixer. Hexanes were then introduced to the elastomers at different ratios: 1:8 for ExSil‐100 and 3:10 for PDMS (40:1 and 60:1) by weight, in order to increase eventual LM droplet size. Eutectic gallium indium alloy (Ga:In, 3:1 by mass) was added to create the LM emulsion ink. The silicone polymer/hexane solution was combined with LM at targeted ϕ (10%, 30%, 50%, or 60%) by volume to create the emulsion, which was further mixed for one minute at 800 rpm to achieve an approximate LM droplet size of 200 μm. The ink was then exposed to a vacuum chamber with a pressure of about 22 in Hg for 3 h in order to remove the hexanes. Lastly, the ink was loaded into a syringe and printed using DIW. The final printed sample was cured in an oven at 80 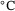 for overnight. A Hyrel Engine SR 3D printer was used to print the LM inks onto fluorinated ethylene propylene (FEP) film for ease of sample removal. Nordson EFD Optimum SmoothFlow nozzles (D = 0.84 mm) were used to extrude the ink. An extrusion velocity of C = 4.1 mm s^−1^ was used for both $V^{*}$ = 2 and 12 prints, while the print head velocity was V = 8.2 mm s^−1^ and 49.2 mm s^−1^ for $V^{*}$ = 2 and 12 filaments, respectively. The crack propagation demo print was created by printing one layer of spherical droplets printed at $V^{*}$ = 2, H = 210 μm and printing three layers of elongated droplets printed at $V^{*}$ = 12, H = 70 μm. For multilayer prints, the print bed was heated to 80 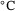 when using PDMS‐based ink to ensure proper deposition on the semi‐cured layers, whereas for ExSil‐based ink, the print bed remained at room temperature. Additionally, each layer was partially cured before printing the next using a heat gun at 130 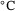 for 3 min. Before each subsequent layer, the extruder was allowed to come to equilibrium via several extruding passes at the $V^{*}$ = 2 or 12.

### 4.2 Rheological Analysis

The rheological behavior of the three ink formulations was characterized using an HR‐30 rotational rheometer (TA Instruments) equipped with 25 mm diameter stainless steel parallel plates. A consistent gap of 0.5 mm was maintained for all measurements. Viscosity profiles were obtained through flow sweep tests over a shear rate range of 0.1 to 10 $s^{-1}$ under ambient conditions (25 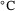).

### 4.3 Image Analysis

To analyze the LM microstructure, such as the alignment angle (θ) and fracture surface angle, optical microscopy images were obtained using a Zeiss Axio Zoom v16 stereo microscope. When measuring θ, we used DIW‐printed samples that were die‐cut into tensile dogbone shapes at specific angles (0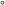, 30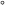, 45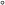, 60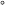, 90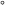). After measuring θ, the absolute value was used, meaning that $+5^{\circ }$ is considered equivalent to $-5^{\circ }$. At least 100 particles were measured to calculate the mean and standard deviation (s.d.). To obtain the fracture surface angle, we used the same samples as those used to measure θ. After breaking the samples through tensile loading using an Instron machine (same method as described in the mechanical characterization section), we measured the fractured surface angle as the angle between the tensile loading direction and the fractured surface. Three samples were measured to calculate the mean and s.d..

### 4.4 Mechanical Characterization

The uniaxial tension tests were performed to determine the sample's tensile modulus with an Instron 5944 mechanical testing machine, and samples were held by pneumatically controlled grips. We used tensile dogbone samples (uniformly scaled to 50% size of ASTM D412‐C) which have a width of 3 mm and thickness of ∼ 0.5 mm. Sample length between the grips was ∼ 35 mm. Samples were strained using a 10 N (for LM composite) or a 50 N (for unfilled elastomer) load cell with a strain rate of 1 mm s^−1^. The tensile modulus was calculated from the slope of the stress–strain curve up to 10% strain. To evaluate mechanical stability of LM composite under cyclic deformation, samples of identical dimensions and the same load cell were employed at a strain rate of 5 mm s^−1^.

### 4.5 Fracture Energy Measurement and Crack Propagation Demo

Acrylic plates with dimensions of 25 mm × 100 mm were used to clamp the samples. Samples were adhered to the acrylic plates using a small amount of cyanoacrylate adhesive (Loctite Superglue) and Sil‐Poxy. The samples with clamps were then left overnight in 40 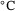 convection oven to allow the adhesive to cure. The final dimensions of the samples after being attached to the clamps was ∼70 mm in width and approx. 15 mm in length. A 20 mm notch was then cut into the sample with a razor blade. The tensile loading for the notched pure shear test was done using the Instron 5944 with a 50 N load cell and strain rate of 1 mm s^−1^. The fracture energy is determined by a Rivlin and Thomas method [65].

### 4.6 Dynamic Mechanical Analysis (DMA)

The viscoelastic properties of the printed LM composites were characterized using a Discovery DMA 850 (TA Instruments) in extensional mode. All measurements were conducted at a constant temperature of 30 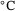. To determine the linear viscoelastic (LVE) region, oscillation strain sweep tests were performed at a frequency of 1 Hz. Stress relaxation behavior was analyzed by applying a constant tensile strain of 1% (within the LVE region) and monitoring the stress decay over a period of 10 min. Creep tests were carried out by applying a constant tensile stress of 0.005 MPa and recording the resulting strain for 10 min.

### 4.7 Crack Tip Analysis

The location of the crack tip was analyzed using Tracker video analysis software by manually marking the location of the crack tip as a point mass in the recorded video of uniaxial mechanical testing of the samples.

### 4.8 Soft Electronics Fracture Demo

The printed sample was adhered to acrylic plates similar to crack propagation demo and LM traces were spray‐coated on the surface of the sample at predetermined location using a laser cut stencil mask. Blue LEDs (DigiKey Electronics) were placed at the LM terminals and encapsulated with uncured ExSil‐100 elastomer. Adhesive copper strips (3 mm width) were attached at the ends of the LM traces, which were then connected to a power supply. A 20 mm notch was made at one end of the sample and tested on a tensile tester using a similar method as the crack propagation sample.

### 4.9 Statistical Analysis

The meaning of all error bars was described within the captions of the corresponding figures.

## Author Contributions

G.M.S., O.H., R.T., E.J.M., and M.D.B. designed research. G.M.S. and O.H. prepared the samples and performed experiments with assistance from R.T. and A.H. G.M.S., O.H, R.T, E.J.M., and M.D.B. wrote and revised the original draft. E.J.M. and M.D.B. acquired funding and supervised the research. All authors discussed the results and commented on the paper.

## Conflicts of Interest

The authors declare no conflicts of interest.

## Supporting information


**Supporting File 1**: adma72447‐sup‐0001‐SuppMat.pdf.

**Supporting File 2**: adma72447‐sup‐0002‐MovieS1.mp4.

**Supporting File 3**: adma72447‐sup‐0003‐MovieS2.mp4.

**Supporting File 4**: adma72447‐sup‐0004‐MovieS3.mp4.

**Supporting File 5**: adma72447‐sup‐0005‐MovieS4.mp4.

## Data Availability

The data that support the findings of this study are available from the corresponding author upon reasonable request.
