# Supplementary material for: Liquids as Reinforcements for Anisotropic and Tough Soft Matter Composites
Source: Adv Mater. 2026 Feb 7;38(33):e72447. doi: 10.1002/adma.72447 (PMC13261386; doi:10.1002/adma.72447)
Supplement: Supplementary file 1 — Supporting File 1: adma72447‐sup‐0001‐SuppMat.pdf. [file ADMA-38-e72447-s004.pdf]

Supplementary Information

Liquids as Reinforcements for Anisotropic and Tough Soft Matter Composites

Gwyneth M. Schloer,[1†] *Ohnyoung Hur*,[1†] *Ravi Tutika*,[1,2†] *Aaron Haake*,[3] *Eric J. Markvicka*,[3,4,5*] and *Michael D. Bartlett*[1,2*]

[1]Mechanical Engineering, Soft Materials and Structures Lab, Virginia Tech, Blacksburg, VA 24061, USA.

[2]Macromolecules Innovation Institute, Virginia Tech, Blacksburg, VA 24061, USA.

[3]Mechanical & Materials Engineering, Smart Materials & Robotics Lab, University of Nebraska–Lincoln, Lincoln, NE 68588

[4]Electrical & Computer Engineering, University of Nebraska–Lincoln, Lincoln, NE 68588

[5]School of Computing, University of Nebraska–Lincoln, Lincoln, NE 68588

[†]These authors contributed equally to this work
[*]To whom correspondence should be addressed: eric.markvicka@unl.edu, mbartlett@vt.edu

Supplemental notes

Following the Cox-Krenchel model modified by Carman-Reifsnider[45], we can see that the elastic modulus of a short fiber composite (E_c) can be described by:

$$E_c = E_i\,\eta_0\,\eta_l\,\phi_i + E_m(1-\phi_i) \tag{S1}$$

where E_i, and E_m are the elastic moduli of the inclusion and the matrix, and ϕ_i is the volume loading of fiber inclusions. η_l is the Cox fiber-length efficiency factor which takes into account the aspect ratio (AR) and relative material properties orientation and η_0 is the Krenchel orientation factor which considers the inclusion orientation.

$$\eta_l = \frac{tanh\,(\beta\,\frac{L}{d})}{\beta\,\frac{L}{d}} \tag{S2}$$

where L is the length, d is the diameter of the fiber, $\frac{L}{d}$ can be written as AR for LM inclusions, and β is,

$$\beta = \sqrt{\frac{-3\,E_m}{2\,E_i\,ln\,\phi_i}} \tag{S3}$$

$$\eta_0 = \Sigma\,a_n\,cos^4\theta \tag{S4}$$

where a_n is the fraction of fibers oriented at a specific angle.

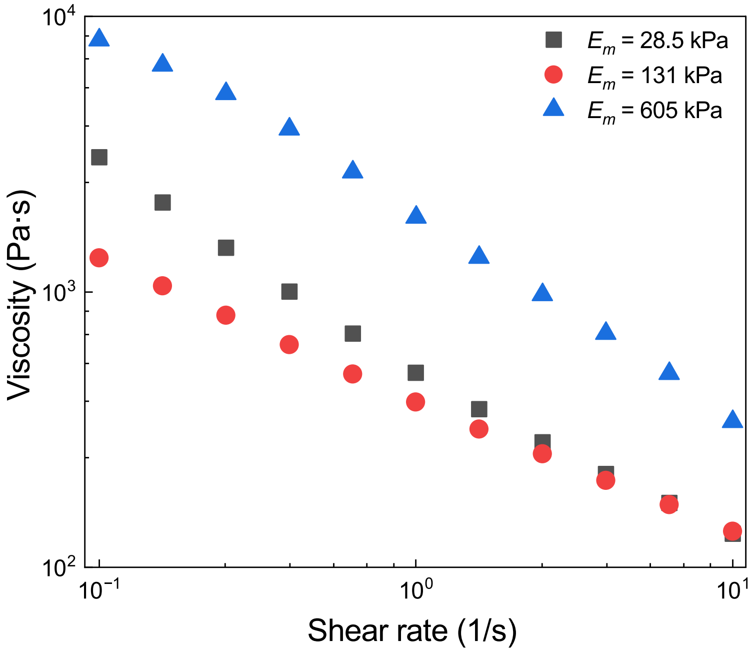

Figure S1: **Viscosity of different modulus-based inks with a fixed liquid metal loading ($\phi = 50\%$).**

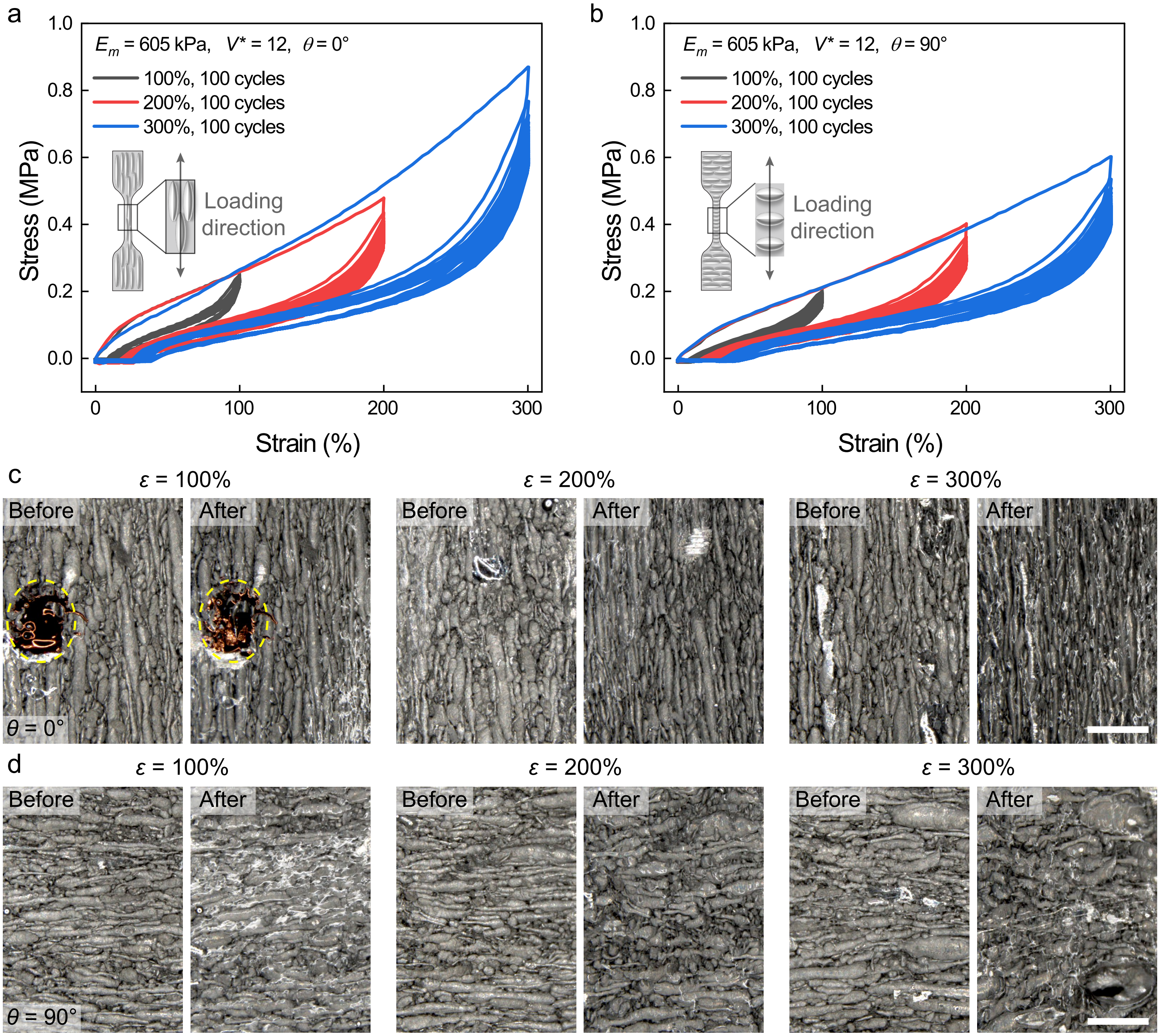

Figure S2: **Cyclic tensile deformation of LM composite (E_m = 605 kPa).** a) Cyclic stress-strain curves for $V^* = 12$, $\theta = 0°$. b) Cyclic stress-strain curves for $V^* = 12$, $\theta = 90°$. c) Micrographs of the LM composite ($V^* = 12$, $\theta = 0°$), before and after 100 cycles at 100%, 200%, and 300% strain. The yellow circle serves as a marker to track the same position. Scale bar is 500 μm. d) Micrographs of the LM composite ($V^* = 12$, $\theta = 90°$), before and after 100 cycles at 100%, 200%, and 300% strain. Scale bar is 500 μm.

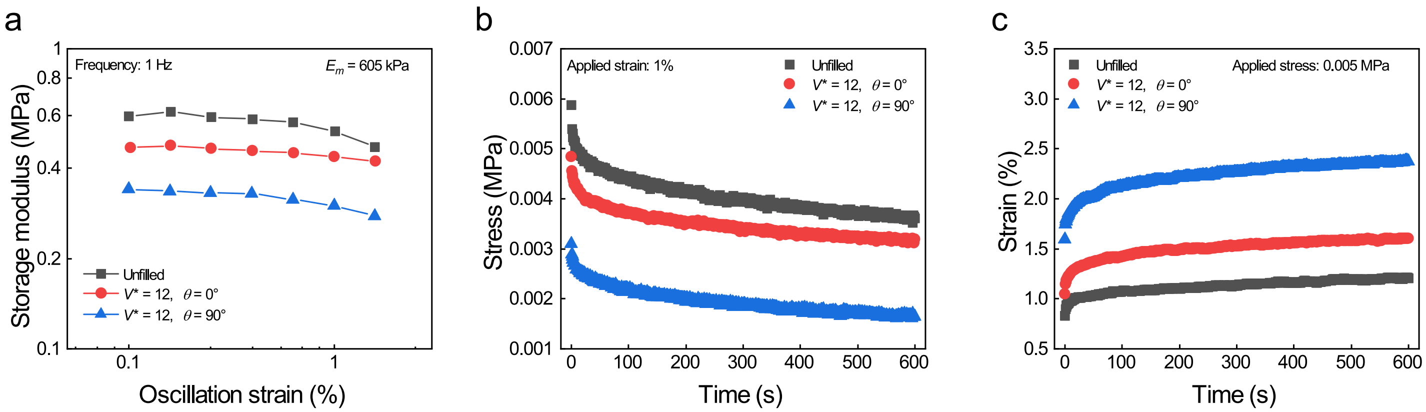

Figure S3: **Viscoelastic characterization of DIW-printed LM composites (E_m = 605 kPa).** a) Amplitude sweep (1 Hz) showing storage modulus vs. strain. b) Stress relaxation at 1% strain. c) Creep strain under 0.005 MPa tensile stress.

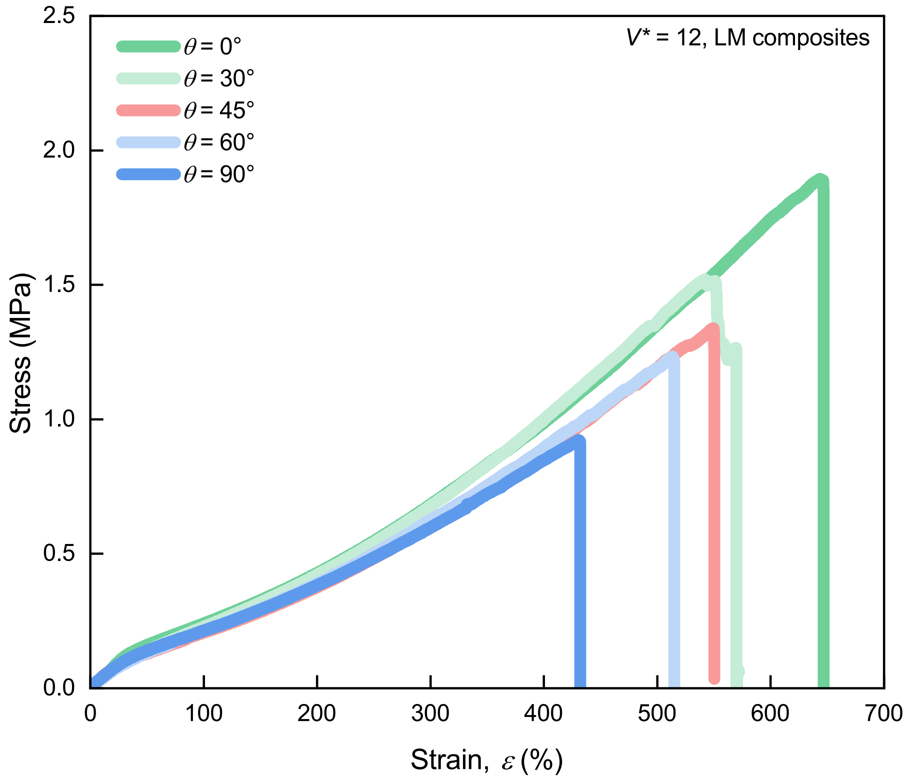

Figure S4: **Stress strain curves of LM composite with $V^* = 12$ with different θ.**

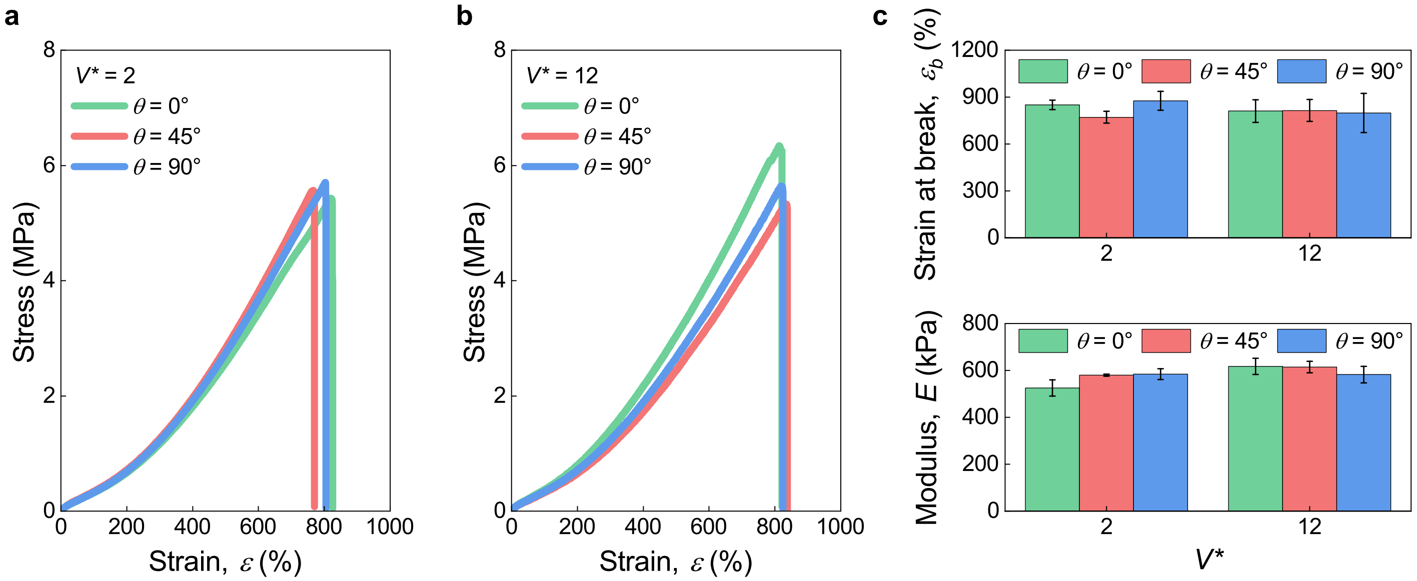

Figure S5: **Mechanical properties of printed unfilled elastomer.** Stress strain curves for unfilled elastomer printed with a) $V^* = 2$, b) $V^* = 12$ and die-cut with θ of 0°, 45°, and 90°. c) Strain at break (ε_b) and modulus (E). Data presented as mean ± 1 s.d. ($n = 3$).

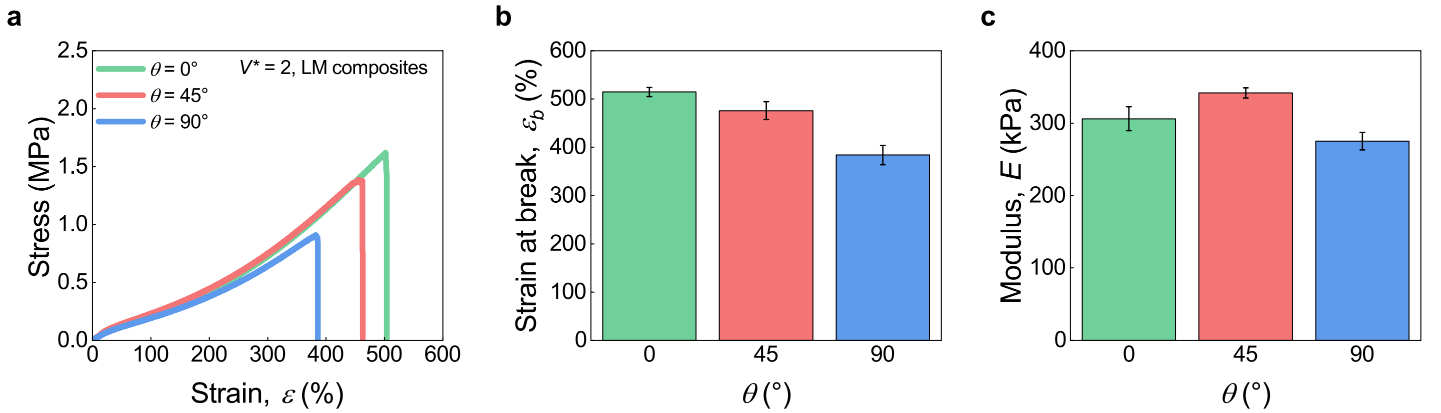

Figure S6: **Mechanical properties of LM composite with $V^* = 2$ and die-cut with θ of 0°, 45°, and 90°.** a) Stress strain curves. b) Strain at break (ε_b). c) Modulus (E). Data presented as mean ± 1 s.d. ($n = 3$).

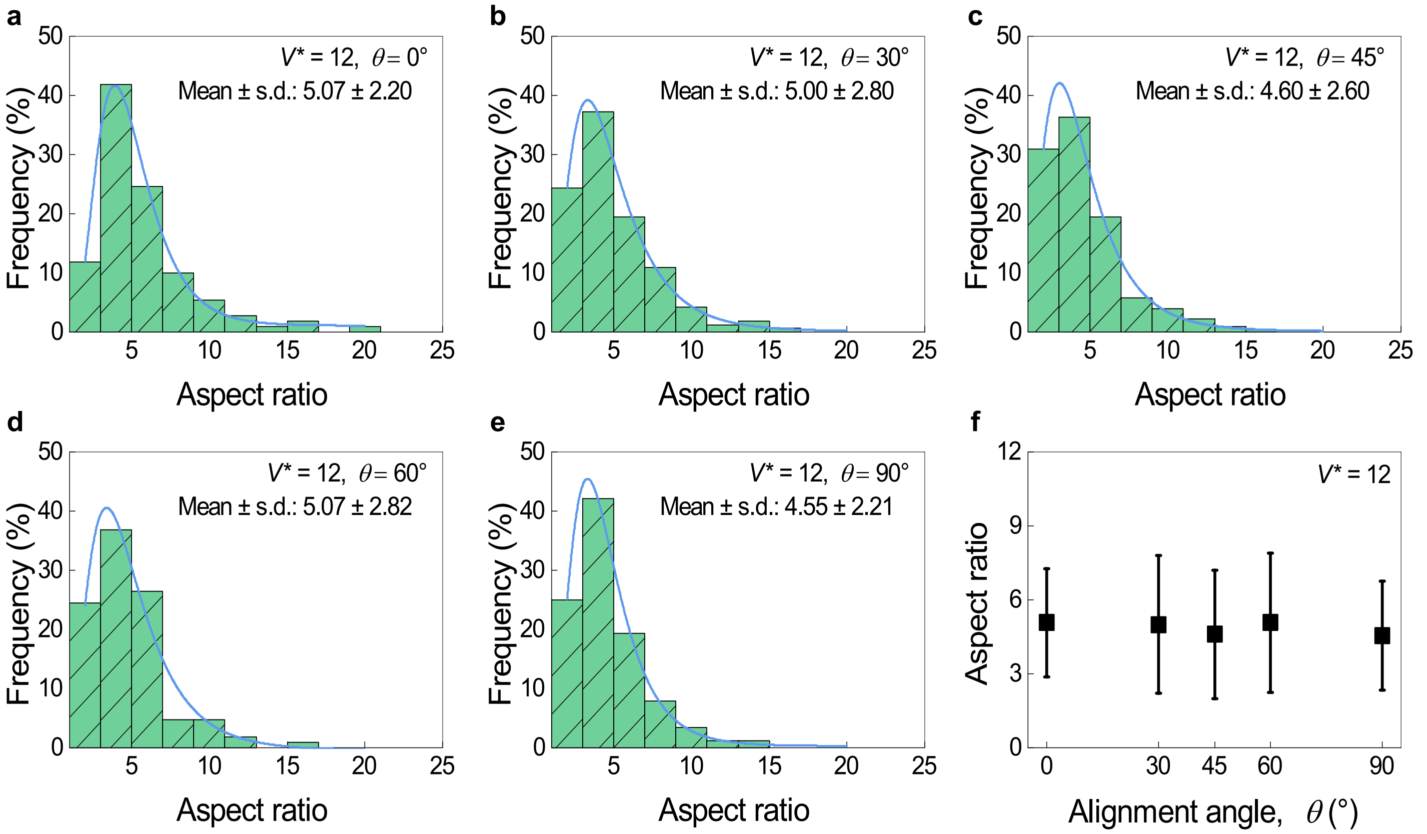

Figure S7: **LM microstructure analysis of LM composite printed with $V^* = 12$.** Histogram of aspect ratio of LM particles in each samples: a) $\theta = 0°$, b) $\theta = 30°$, c) $\theta = 45°$, d) $\theta = 60°$, and e) $\theta = 90°$. At least 100 particles were analyzed with log-normal fit (solid blue line) by using Origin software. f) Mean value of aspect ratio with different die-cut angle. An average of the five mean values ($AR = 4.90$) is taken for the short-fiber model fit in Fig. 2c. Error bar is s.d. from log-normal fit.

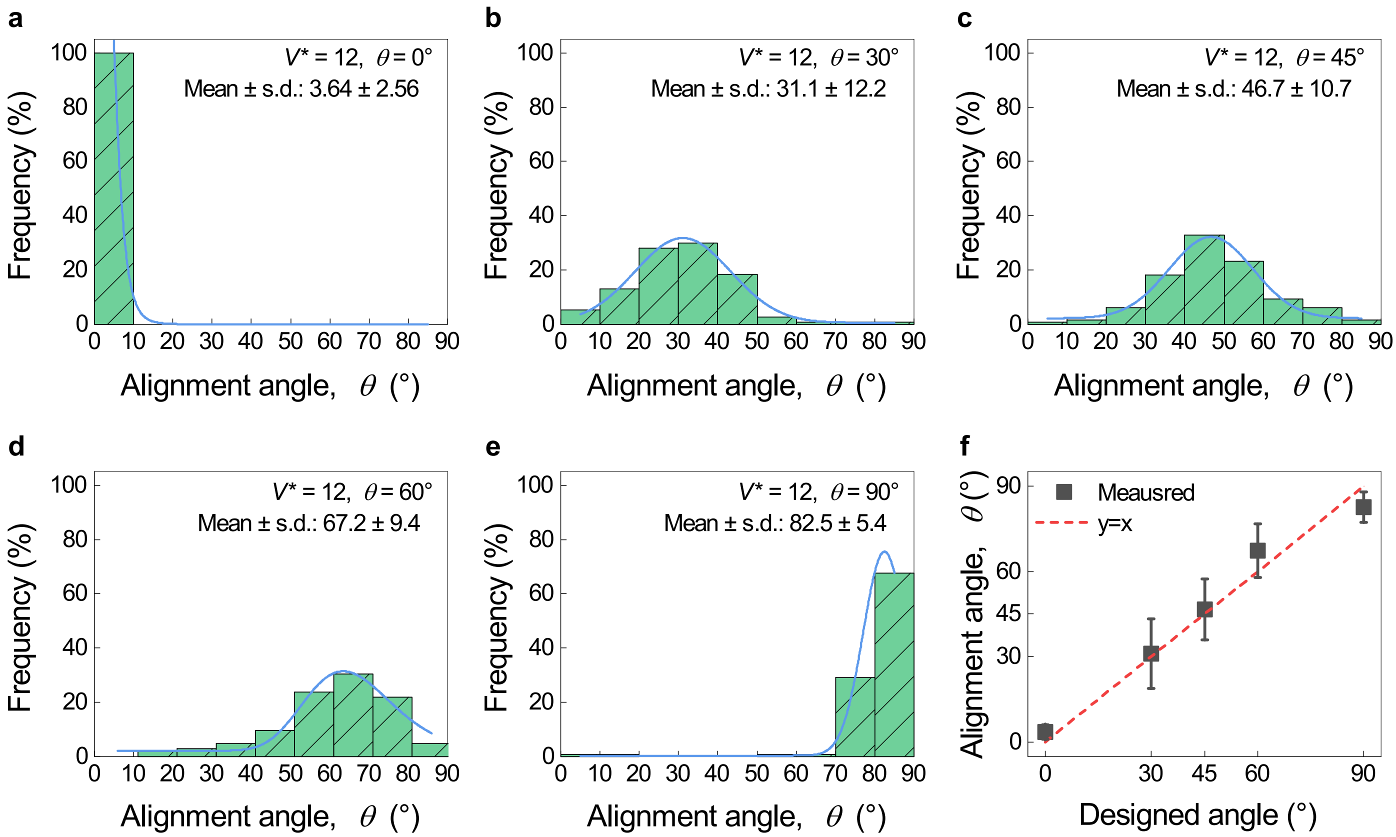

Figure S8: **LM microstructure analysis to understand anisotropy in LM composite printed with $V^* = 12$.** Histogram of alignment θ with different die-cut angle of a) $\theta = 0°$, b) $\theta = 30°$, c) $\theta = 45°$, d) $\theta = 60°$, and e) $\theta = 90°$. At least 100 particles were analyzed with log-normal fit (**a** and **e**, solid blue line) and Gaussian fit (**b**, **c**, and **d**, solid blue line) by using Origin software. **f,** Measured alignment angle (θ) vs. designed angle. The error bar is s.d. from log-normal and Gaussian fit.

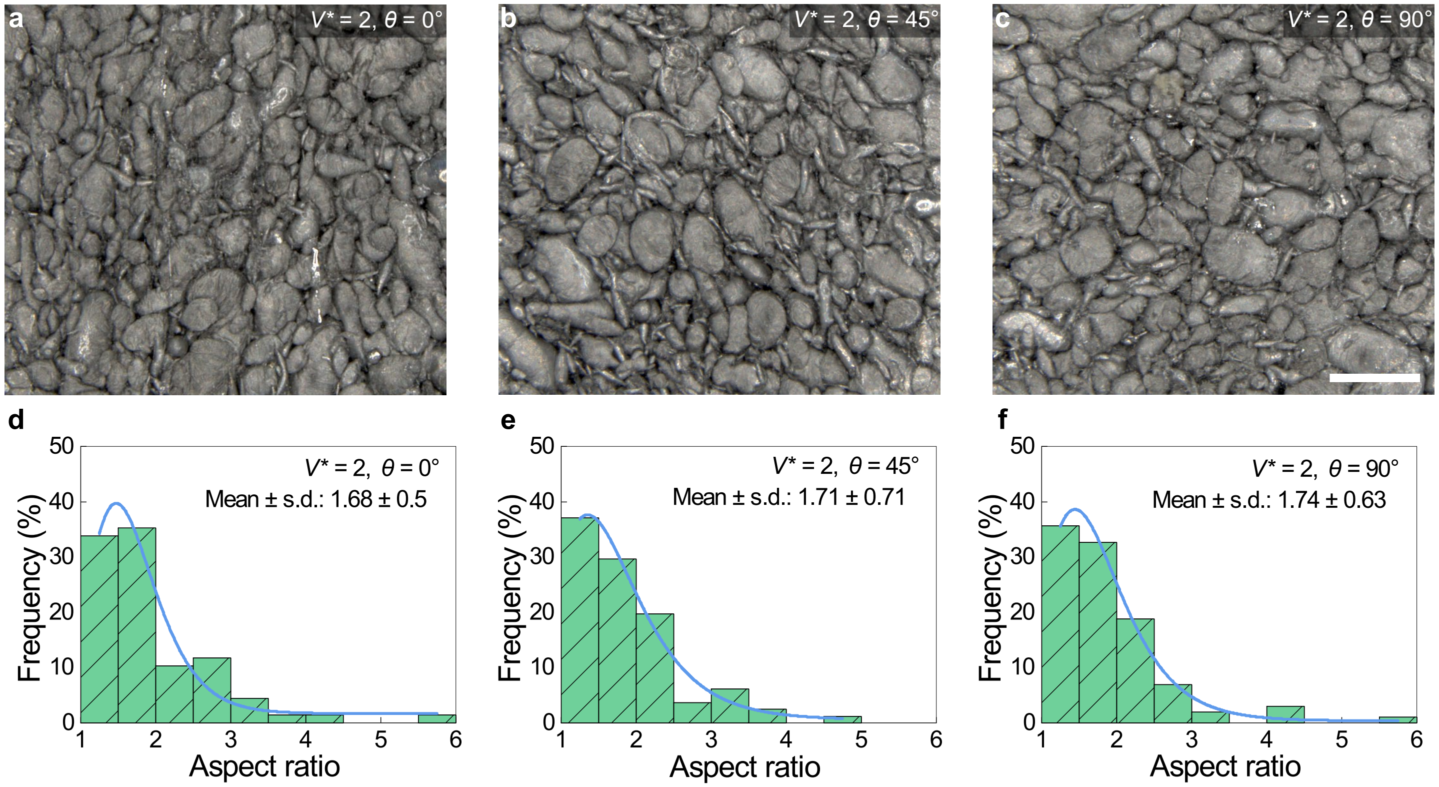

Figure S9: **LM microstructure analysis of LM composite printed with $V^* = 2$.** Microscopic images: die-cut with θ of a) 0°, b) 45°, and c) 90°. Scale bar is 500 μm. Histogram of aspect ratio of LM particles in each sample: d) $\theta = 0°$, e) $\theta = 45°$, and f) $\theta = 90°$. At least 100 particles were analyzed with log-normal fit (solid line, blue) by using Origin software.

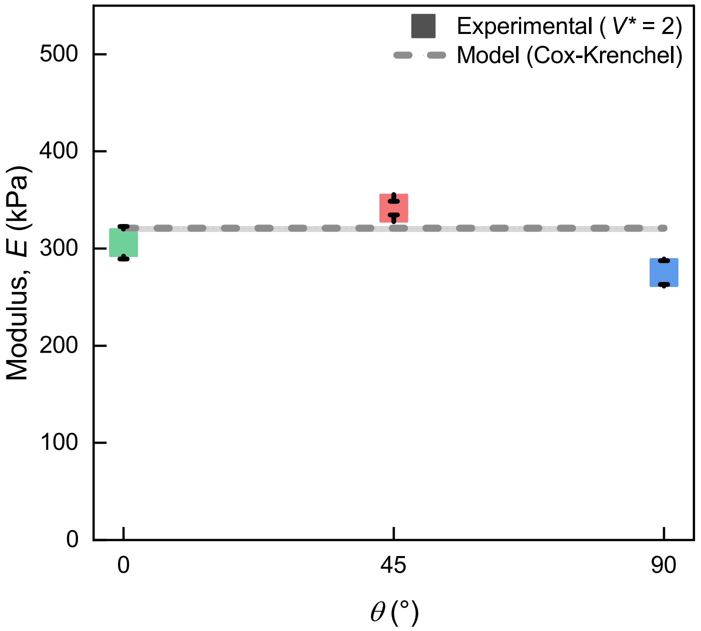

Figure S10: **Cox-Krenchel model fitting** LM composite printed with $V^* = 2$. η_0 for this is fixed as 0.2 as it does not have any orientation. Data presented as mean ± 1 s.d. ($n = 3$). The dash line is the model fit with the mean aspect ratio (AR) and the gray shadow is the error from the s.d..

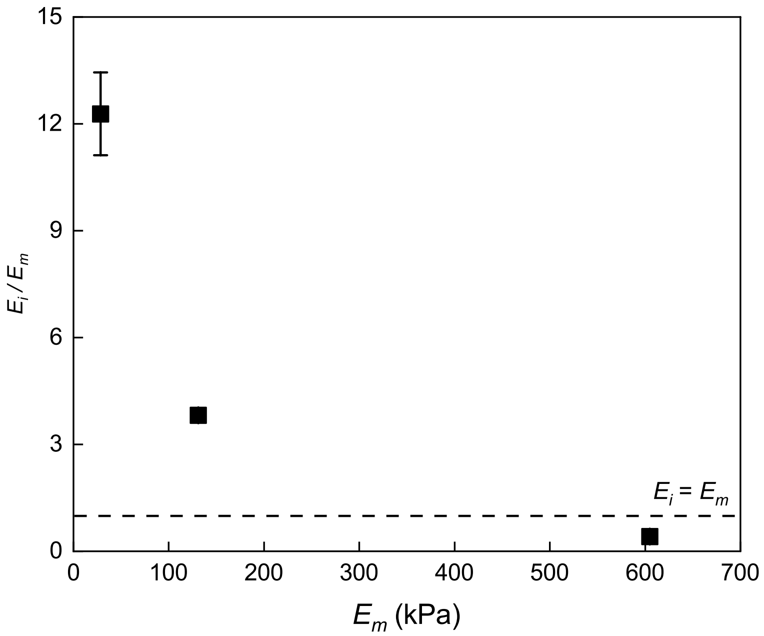

Figure S11: **Modulus ratio between inclusion (E_i) and matrix (E_m).** Data presented as mean ± 1 s.d. ($n = 3$).

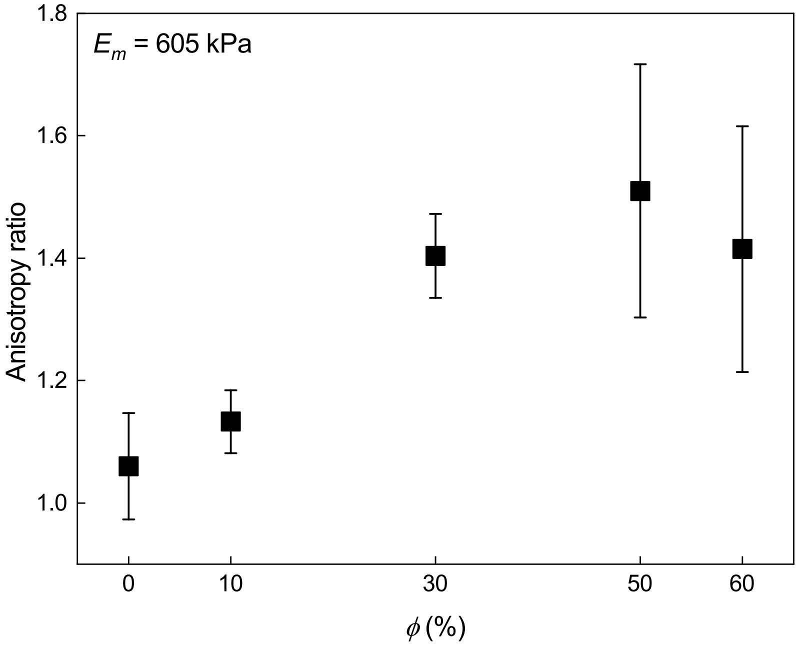

Figure S12: **Anisotropy ratio of LM composite as a function of liquid metal volume loading (ϕ).** The matrix modulus (E_m) is maintained at 605 kPa for all samples. Anisotropy ratio represents the ratio of the modulus at 0° to that at 90° ($E_{c,\,\theta=0°}/E_{c,\,\theta=90°}$). Data are presented as mean ± 1 s.d. (n = 3).

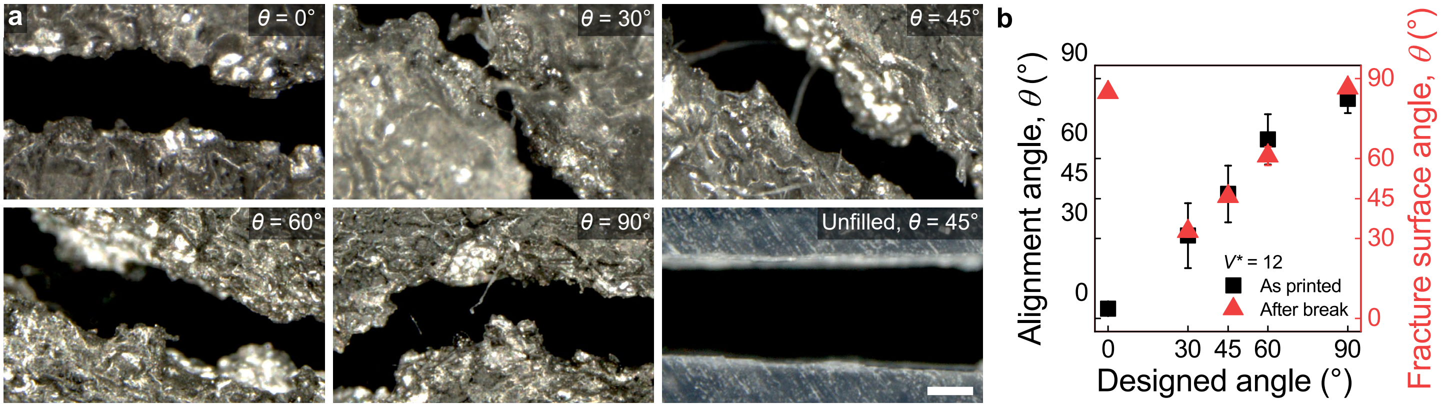

Figure S13: **Microscopic images of broken dog-bone sample after tensile testing.** a) Optical micrographs of LM composite tensile samples ($V^* = 12$, $\theta = 0°$, $30°$, $45°$, $60°$, $90°$), and an unfilled elastomer tensile sample ($V^* = 12$, $\theta = 45°$) after break. Scale bar is 500 μm. b) Plot of alignment angle and fracture surface angle of LM composite ($V^* = 12$) with respect to the designed angle. Data presented as mean ± 1 s.d. ($n = 3$).

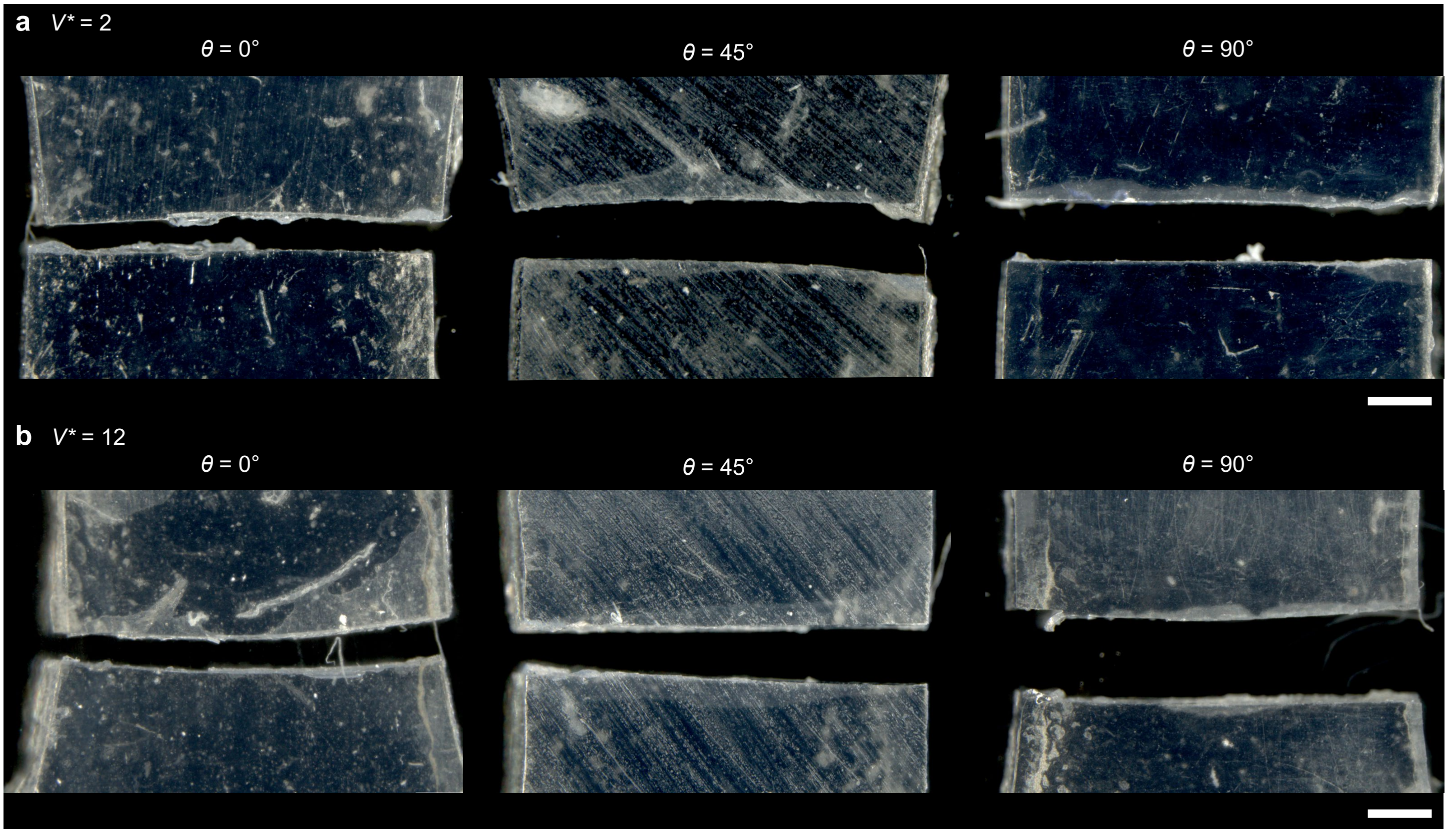

Figure S14: **Microscopic images of broken dog-bone sample after tensile testing.** a) Unfilled elastomer printed with $V^* = 2$ and die-cut with θ of 0°, 45°, and 90°. b) Unfilled elastomer printed with $V^* = 12$ and die-cut with θ of 0°, 45°, and 90°. Scale bar is 500 μm.
